# Ethical decision making in airway management: a Difficult Airway Society position statement on good practice

**DOI:** 10.1016/j.bjao.2025.100416

**Published:** 2025-01-19

**Authors:** Barry McGuire, Simon Crawley, Nicki Dill, Paul Greig, Rehana Iqbal, Mathew Patteril, Kate Rivett, Anika Sud, Anne-Marie Slowther

**Affiliations:** 1 Ninewells Hospital and Medical School, Dundee, UK; 2 Barema, London, UK; 3 Guy's and St Thomas' NHS Foundation Trust, London, UK; 4 Department of Anaesthesia, Mediclinic City Hospital and Mohammed Bin Rashid University, Dubai, UAE; 5 University Hospitals of Coventry and Warwickshire, Coventry, UK; 6 DAS Lay Representative, London, UK; 7 Oxford University Hospitals NHS foundation Trust, Oxford, UK; 8 Warwick Medical School, University of Warwick, Coventry, UK; 9 Nuffield Department of Clinical Neurosciences, University of Oxford, Oxford, UK

**Keywords:** airway, consent, ethics, innovation, training

## Abstract

Practitioners involved in airway management must balance ethical issues in their practice. Ethical tensions exist because clinicians must maintain clinical standards while maximising skill development, exploring advances in airway practice, and incorporating new learning to benefit future patients. Balancing the benefits and risks to the patient and choosing the right techniques in the right situations and with the right level of patient understanding and respect for patient autonomy can be challenging.

These challenges are shared by airway practitioners from many professional backgrounds; however, this document has been developed specifically to support anaesthetists in their airway management decisions, and for simplicity, the term ‘anaesthetists’ will be used throughout the document. However, the ethical considerations will have relevance to all airway practitioners.

Practice combined with training is central to professional development. Most patients are aware that training is entwined with care and trust anaesthetists to deliver this safely. Trainers should use airway teaching methods appropriate to the trainee's needs and skills.

Informed consent is required for airway management, and the level of detail should be proportionate to the risks involved. Patients have individual preferences and appreciation of risks, so these conversations must be individualised.

Anaesthetists should support the development of new airway devices and techniques. New methods must be assessed within governance structures, and it may be appropriate to collect data or feedback as part of the introduction to practice.

Ethical practice requires doing what is best, doing it openly, honestly, and in patients' interests. The modern ethical and legal landscape has emphasised patient information, discussion, and documentation. We hope this position statement provides guidance, structure, and clarity for the benefit of our patients and our specialty.

## Introduction

The practice and learning of airway management can create ethical challenges, notably around risks and benefits, and how these are addressed when seeking informed consent. There are no existing guidance documents on this specific topic. However, generic guidance on practice—given in the General Medical Council (GMC) documents on *Good Medical Practice* (2024), *Decision Making and Consent* (2020), *Confidentiality* (2017), and *Disclosing Information for Education* (2017)—is relevant.^1^, ^2^, ^3^, ^4^ Consent for anaesthesia should include reference to airway management, and there are several factors which may make this more challenging: (i) airway management is not generally well understood by patients^5^; (ii) it is not usual practice for most anaesthetists to have detailed conversations regarding airway management, perhaps beyond discussion of common risks^6^, ^7^, ^8^; and (iii) patients may be less receptive to a discussion regarding procedural aspects of their anaesthesia and the associated risks. These factors may create a disconnect between what currently happens in airway management practice and ideal informed consent.

Airway management requires choice of both endpoint and technique. There may be several options in terms of endpoint, including oral or nasal tracheal tube, supraglottic airway device, or tracheostomy. The technique used to reach that endpoint similarly varies. Each combination of device and technique will have a differing risk–benefit profile with choices being made through appraisal of risk profile, and discussion with the patient. In certain clinical situations, there will be an obvious choice (e.g. using a tracheal tube in a patient with a full stomach or a nasal flexible bronchoscopic intubation in a patient with trismus) and, in others, clear risks (e.g. nasal intubation in an anticoagulated patient). Patient choice may force a clinician into considering a higher-risk option. A patient's wish to avoid tracheostomy or an awake intubation, for example, may add risk and the consequence of that risk must be communicated through informed consent.

The risk–benefit assessment is often nuanced and requires professional judgement. There may be multiple options to consider, and local practice, culture, and experience are relevant. This contrasts with other aspects of anaesthesia, such as spinal/epidural procedures, where techniques and equipment are more standardised.

Major complications of airway management, although rare, cause significant morbidity and mortality. Problems with tracheal intubation were the most frequent major airway complications in both the 4th National Audit Project (NAP) report and the American Society of Anesthesiologists Closed claims analysis in 2011.^9^^,^^10^ Airway complications are linked to the poorest outcomes and highest costs in UK litigation claims relating to anaesthesia,^11^ bringing potentially life-changing consequences for a patient, or even death.^12^ Contributing factors relate to self (stress, fatigue, inadequate training), clinical context (staff, patient, equipment, location, timing) and task (task overload, distractions). Many of these are predictable and may have been controllable or avoidable. A more ethical mindset, that fully appraises potential harms through application of foresight, may enable a more complete and pre-emptive identification of risk when undertaking airway management.^13^ The UK Supreme Court judgement in *Montgomery v Lanarkshire Health Board* (2015) highlighted the importance of material risk disclosure during consent conversations and this is an essential element of patient-centred care.^14^

The important underlying truth to all the above is that the duty to protect patients from harm is a foundational ethical principle in the health care system in which we work. In 1979, the four principles of biomedical ethics (respect for autonomy, beneficence, non-maleficence, and justice) were proposed by Beauchamp and Childress^15^ and continue to underpin clinical practice today. The principles require doctors to always act with integrity and honesty, to treat patients fairly, maximising benefits and minimising harms, to involve patients in decisions about their care, and to respect their choices. These principles are reflected in the GMC's *Duties of a Doctor* guidance from the National Institute for Health and Care Excellence (NICE) and influence key legal judgements.^1^^,^^14^^,^^16^ Modern medicine has moved away from paternalistic doctor–patient models to those emphasising shared decision making.^2^^,^^16^ Airway management should reflect this.

Airway skills must be acquired and mastered as anaesthetists develop from novice to expert, and technical skills alone are not sufficient. The experienced airway provider must also bring ethical judgement to their practice. Innovation in equipment and techniques means that practice continually evolves, so anaesthetists must develop and maintain new skills through learning. Experienced colleagues must support others through teaching and training. These activities need to take place within governance, regulatory, and research frameworks.

The challenge for anaesthetists is how to balance these duties when considering (i) the current patient being cared for; (ii) advancement of the specialty and the interests of future patients; and (iii) the wider societal context of changing patient expectations, reflected in the medico-legal landscape. Historically, there has been considerable inconsistency in how clinicians approach this balance.

There is a potential ethical tension between the development of a clinician's clinical skills and their professional responsibility to prioritise the safety of their patient.^1^^,^^17^ This is particularly relevant to airway management, where there may be several alternative approaches of varying familiarity to the anaesthetist, with risks potentially uncertain or difficult to quantify. There is natural variation in skills and experience between anaesthetists, and no one can master every possible combination of technique and approach, but anaesthetists should achieve competence in a range of airway techniques to ensure that they have options to draw on.^5^ This learning has always taken place in parallel to the provision of patient care and this should continue. However, clinicians and educators need to be cognisant of the ethical principles involved in the process of teaching and learning airway skills with patients.

Research and innovation in airway management, within well-established governance structures, are integral to the development of the speciality. This contributes to improved care for future patients. However, informal attempts to introduce new devices outside of proper governance structures must be discouraged. Improper adoption of new techniques puts patients at risk of harm from device or technique failure. Complications may arise from deficiencies in training or unfamiliarity with the new system. Opportunities to gather data to inform the profession may be lost. Clinicians must therefore ensure that the introduction of new devices and techniques is performed with consideration of the available evidence, appropriate educational support, data collection, and, if formal research is being undertaken, within ethical and regulatory frameworks.

Given the current lack of specific guidance, a working group was established to review ethical decision making in airway management. The resulting document aims to provide practical recommendations, as outlined in Table 1, for providing ethical practice when choosing airway techniques, delivering training, introducing new airway devices, and collecting clinical or research data. It does not constitute legal advice and relates to airway management in adults and does not address the specific ethical or legal frameworks for treating children. It is written for UK anaesthetists, their departments, and their trusts, although it may have relevance in other countries. We also hope that non-anaesthetists who manage the airway, notably emergency medicine and critical care physicians, will find useful information here. This document draws on generic guidance provided, for example, by the GMC and tailors it to airway management decisions.

**Table 1:** Recommendations for airway managers for the provision of ethical clinical practice, training, equipment implementation, and information sharing.

1. Anaesthetists and all other airway managers must practice ethically, showing respect for patient autonomy and beneficence for every patient. 2. Airway management should be tailored for each patient regarding the choice of device/technique and, when appropriate, to facilitate training. Informed consent is necessary. 3. Ethical checklists and frameworks should guide the approach to airway management. 4. Anaesthetists should optimise preoperative information sharing, risk–benefit discussion, and consider using a structured approach to patient conversations regarding airway management. 5. The introduction of new airway devices/techniques within a department must involve explicitly informing clinicians about key features, rationale for use, and any need for training. 6. Teaching airway management should reflect the learner's stage in training, their level of competence, and their anticipated need for the taught skill. Airway teaching during routine lists should be referenced as part of preoperative informed consent. 7. Anaesthetists should promote the progress and understanding of safe and effective airway management by supporting rigorous formal investigation of new devices and techniques. 8. Airway management research and innovation must be performed within the local and national regulatory frameworks of research ethics. All forms of patient data collection, regardless of project design, must be conducted within an appropriate governance framework. 9. Anaesthetists should support device procurement processes in accordance with professional, legal, and ethical standards—both local and national—including reference to device performance data and the provision of appropriate training before its wider clinical availability.

## Objectives

The working group considered key objectives when formulating their recommendations as shown in Table 1:

1. To consider the specific ethical issues and challenges relating to airway management.
2. To provide practical advice on applying general ethical principles to decision making and encourage clinicians to engage patients in conversations relating to risks, benefits, and informed consent within airway management.
(Recommendations 1, 2, 3, and 4)
3. To provide guidance on tailoring training to the learner and to encourage training, skill acquisition, and education to be part of informed consent.
(Recommendations 5 and 6)
4. To highlight the importance of adherence to ethical and governance standards when undertaking airway-related projects or research; handling patient data; and when evaluating, procuring, or implementing new equipment.
(Recommendations 5, 7, 8, and 9)

## Methods

A working group was formed under the guardianship of the Difficult Airway Society (DAS), chaired by its President. The group included anaesthetists with interest or expertise in airway management and medical ethics, a professor of clinical ethics, the chair of the Association of Anaesthetic and Respiratory Device Suppliers (BAREMA), a lay representative, and representation from both the Royal College of Anaesthetists (RCoA) and The Association of Anaesthetists (AoA). An exploratory review of relevant literature identified mainly opinion papers or legal reports. Broad areas within airway management for individual consideration were agreed—namely, ethical issues within clinical practice, teaching, research, and new device introduction. The group met over a 4-yr period to formulate their recommendations through an iterative deliberative process. DAS UK members (trainees and consultants), as representatives of UK anaesthetists, were surveyed to ascertain their views on a variety of ethical scenarios relating to airway management and the findings were considered. Members of the PatientsVoices@RCoA (formerly the RCoA Lay Committee) took part in a guided discussion of the ethical issues raised in the survey. The expressed views and key messages from this discussion are summarised in the paper and incorporated into its development. Additional expert (airway and ethics) review contributed to further refinement of the text (see Acknowledgements). Before submission for publication, we sought review of this paper from the DAS Executive.

## Overview

We first consider the key ethical issues and challenges for anaesthetists in airway management—providing a reasonable standard of care to patients, balancing risks and benefits when selecting an airway management plan, and respecting patient autonomy through informed consent and shared decision making. We then consider how these duties inform two key areas of practice that contribute benefit beyond the individual patient, namely, innovation and training in airway management. Finally, we consider how best to inform and involve patients in decision making about their airway management.

## Key ethical issues and challenges

### The duty to provide a reasonable standard of care in airway management

There is both an ethical and a legal requirement to provide a reasonable standard of care as a minimum. In UK law, the common legal standard for a reasonable standard of care is the *Bolam* test,^18^ that a competent doctor of similar experience would deliver the same care in the same position. The doctor will not be in breach of duty if a responsible body of medical opinion would consider the treatment reasonable, even if other experts might disagree. The *Bolitho v City and Hackney Health Authority* (1998) judgement further qualifies that the decision must be capable of withstanding logical analysis, namely, that the action was defensible.^19^

Anaesthetists have a professional responsibility to provide safe and effective airway management for their current patients. In elective situations, a lack of expertise in a required technique should prompt attempts to find a more appropriately skilled colleague or, if necessary, to postpone a case. In life-threatening emergencies, however, there remains a responsibility to provide care to the best standard available. This could require an anaesthetist practising beyond their specific area of expertise. Examples might include the need for paediatric airway management or for awake tracheal intubation by clinicians who do not routinely use such skills.

Doctors have a professional and legal obligation to train and provide training for the benefit of their future patients.^1^^,^^20^ Airway management can be provided by a trainee, with appropriate supervision. Training and teaching are inseparable from the delivery of patient care and both didactic and experiential learning occur seamlessly. This is as true for those maintaining existing skills as for those adopting new, potentially less familiar devices or techniques. It is important that patients are aware that training is part of their care and that this is supervised, tailored to the learner, and clinically appropriate.

### Balancing risks and benefits in selecting the airway management plan

Anaesthetists must choose airway devices and techniques consistent with providing reasonable care.^21^ Who benefits from the choice can be considered from three different perspectives—the current patient, future patients, or the clinical team. Ideally, the approach chosen will benefit all three, but the current patient takes primacy.^1^

Often, the chosen combination of device and technique is considered standard or *routine* care. Sometimes the choice will be one of several available techniques with the same endpoint and similar risk–benefit profiles. An example would be oral intubation where a variety of laryngoscopes may be used by an individual equally proficient in their use. All could be considered ethically acceptable.

**Fig 1:**
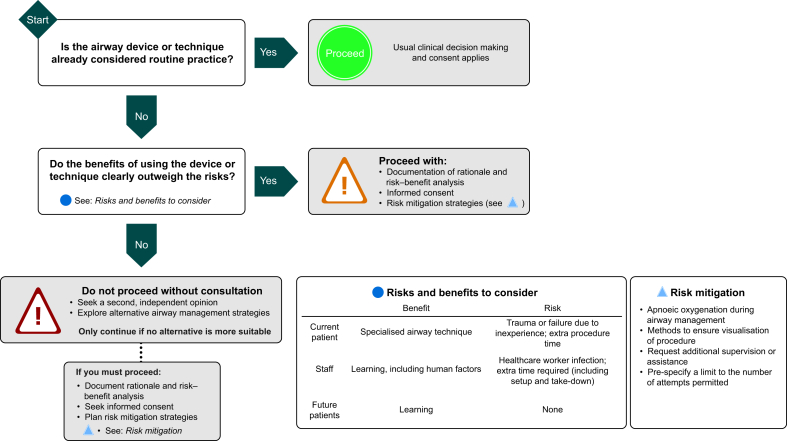
A flowchart to guide the choice of an appropriate airway device and technique.

An airway device, or perhaps more commonly an airway technique, could be deemed *non-routine* if most practitioners would not consider it common practice in their institution, but its use may be ethically justifiable if it offers benefit to the current patient. Specialist skillsets must be given consideration here—an expert who regularly uses an advanced airway technique, such as high-pressure source ventilation, will consider it to be routine, whereas others, less experienced individuals, would not. Non-routine airway approaches may also provide wider benefits to the clinical team or to future patients through education, research, or device evaluation. If the practitioner wishes to move from a routine to a non-routine method, as determined by skillsets within their own individual practice, this should involve additional patient information during the consent process, particularly if this non-routine approach is primarily to benefit others. An example would be use of a conduit intubation through a supraglottic airway device as the primary airway strategy. This is an important airway rescue technique requiring practice and its performance for training purposes should be discussed with the patient. Risk mitigation strategies that may be used during non-routine airway management or during teaching are listed in Table 2, whereas Fig. 1 provides a flowchart guide to choosing an appropriate airway device and technique.

**Table 2:** Risk mitigation strategies during non-routine airway management.

• Ensuring adequate preclinical skill and knowledge, such as manikin practice • Optimising Human Factors and Ergonomics^22^ such as effective team performance, flattening of hierarchies, optimal theatre layout, and use of robust standard operating procedures • The delivery of nasal oxygen during awake or anaesthetised airway instrumentation • Maximising supervisor visualisation (such as using a Macintosh VL to teach direct laryngoscopy) and limiting the number of attempts^23^ • Verbalising pre-agreed triggers at which the non-routine approach will be abandoned

### Respecting patient autonomy

#### Facilitating informed consent

Recent Supreme Court judgements have clarified legal expectations around the consent process.^13^^,^^24^ Doctors must ‘take reasonable care to ensure that the patient is aware of any material risks involved in any recommended treatment and of any reasonable alternative or variant treatments’. Materiality is: ‘ … whether a reasonable person in the patient's position would be likely to attach significance to the risk, or the doctor should reasonably be aware that the particular patient would be likely to attach significance to it’.^14^ A ‘reasonable option’ is one supported by a reasonable body of medical opinion.

Informed consent for anaesthesia stands alongside, but is separate to, surgical consent. It should include discussion of the treatment proposed and *reasonable* alternative treatments, and the potential benefits and risks of each option, including the option of doing nothing.^2^ Although anaesthesia and surgery are intrinsically linked, the option of alternative anaesthetic techniques or doing nothing means a separate conversation must be had. Issues raised here may re-open the surgical discussion. Although potentially challenging, it is a clinician's duty to address these options. Consent should be supported by timely and accurate patient information,^21^ and in the elective setting, this should occur on a separate occasion before the preoperative visit. This information should include reference to the risks of anaesthesia, including airway management. Documentation of the verbal informed consent is recommended. This will usually be concise but occasionally will involve a more detailed record of the discussion, such as risks or alternative approaches.

Achieving this standard for airway management requires consideration of several factors:

(i) Most practitioners choose not to discuss life-threatening sequelae of airway management with patients considered to be at ‘low risk’ (A. Kapila, personal communication, 2018). By legal standards, a basic risk discussion should include the risk of life-threatening sequelae however unlikely. This can be expanded upon in the presence of patient or procedural risk factors and patient preferences for information disclosure, or not discussed if the patient states this preference. A decision to use a non-routine airway technique, outwith any individual practitioners specialist skillset, requires an explanation of why this technique is being proposed.
(ii) There is a wide spectrum of what a patient might want to know about their airway management, ranging from someone who wishes to know nothing through to someone who wants to know every detail of their airway management technique, including complications and consequences of the different airway options. The AoA consent for anaesthesia guidelines (2017) and the GMC guidance on consent recognise that if a patient insists that they do not want to know about the risks of anaesthesia, then the patient should understand there may be risks, but not have a detailed explanation forced upon them.^2^^,^^25^ This approach was supported by discussion with PatientsVoices@RCoA. Anaesthetists must gauge the level of information their patients wish to have and tailor their conversations accordingly.
(iii) Airway management encompasses a spectrum of complexity. On the basis of current legal and ethical guidance, it would seem justifiable to tailor the risk discussion based on the patient and procedural risk profile.
(iv) Airway training and the introduction of novel airway devices or techniques may also need to be addressed during the consent process. These are considered later in this paper.

#### Interpreting and communicating risk

Risk is a complex concept, inseparably linked to individual perception. A risk can be measured by a combination of the probability of a negative event occurring (perhaps known to the anaesthetist) multiplied by the magnitude of its impact (which may only be truly judged by the patient). For risk to be understood, it requires informative dialogue between anaesthetist and patient as both perspectives are required. ‘Material risk’ will differ between individuals as the impact of a negative outcome will have differing consequences between individuals, compounded by the subjectivity of risk perception. Furthermore, the probability of a complication occurring in a particular circumstance is often unknown. Quoting incidences of risk extrapolated from large data studies such as NAP 4, although helpful to a degree, will not directly apply to any individual clinical situation with differences in anaesthetist skill level, patient factors, situational variables, and the airway devices or techniques used. Historical data may not be available or interpretable when a new method is adopted. Basing risk perception on personal experience, practice, and judgement is also flawed as personal biases may influence accuracy.

The significance of a particular outcome to a patient is highly subjective. Disclosure of all variables influencing the risks to which the patient is exposed may be unfeasible, cause confusion and anxiety, or be impossible where insufficient data exist. Even where data exist, for example, the reduced incidence of intubation complications associated with the use of videolaryngoscopy (VL) over direct laryngoscopy (DL),^26^^,^^27^ it can take time for routine practice to adapt to new findings.

#### Shared decision making and patient choice

The *Montgomery* judgement requires that all reasonable options are discussed with patients to help them make a decision.^13^ In airway management, there may be multiple methods available. As with any treatment, anaesthetists should assess what the patient considers material, and tailor their conversation over risks and benefits to meet their needs. This conversation may cover both the endpoint of airway management and the technique used to achieve the endpoint. Such discussion will also be influenced by the complexity and urgency of the clinical situation. It is also important for anaesthetists to be aware of their own potential biases and to be transparent when seeking informed patient consent.

Non-routine airway management should normally involve more explicit patient discussion, so the patient understands the reasons why such an approach is being proposed. For example, an anaesthetist proposing an awake tracheal intubation in a patient, where this would be perceived as the safest technique, would often still consider the technique as ‘non-routine’ and so merit more explanation, although this will be influenced by their own experience and skill in the technique. A patient may occasionally refuse awake tracheal intubation and only consent to airway management during general anaesthesia. The harm of refusing to proceed and delaying the procedure must be balanced against anticipated procedural risks if they proceed as the patient requests. In the elective situation, the anaesthetist cannot go against the patient's wishes but may also refuse to deliver what they deem to be a less safe technique, namely, conventional anaesthesia. It would be advisable to seek a second opinion in this case. In emergency scenarios, this may not be an option and any delay in action may increase the risk to the patient. Furthermore, informed consent may be impossible and airway management should be performed in the patient's best interests as judged by the airway team.

#### Patients who lack capacity to consent

When a person lacks capacity to make decisions for themselves, and where no one has the authority to make decisions on their behalf or the urgency of the situation makes it impossible, the clinician has an ethical and legal duty to act in the person's best interests.^28^ This applies when planning airway management, and where time permits, the anaesthetist should consult those closest to the patient.

Consideration of benefit to others, for example, in the context of training or using a novel technique, would not normally be a legitimate factor when determining best interests and training benefits should not take primacy over those interests of patient care. We acknowledge, however, that there are some specific emergency airway scenarios where it would never be possible to gain experience with patients who can consent, such as intubation during cardiac arrest or major trauma. In such situations, more common in emergency medicine and critical care, training may be consistent with ethical practice but requires careful senior consideration and experienced supervision and should only be delivered at an appropriate stage of the learner's training.

## Innovation in airway management

New methods of airway management enter clinical practice continually. Safe adoption of new methods may need support from innovation, quality improvement, research ethics, and/or clinical governance structures.

### Current standards and regulations

Since the adoption of the Eu-MDR statute in 2017 (European Medical Devices Regulations–EU/2017/745), the medical devices industry has become a highly regulated sector.^29^ Regulation and product requirements ensure certified devices are safe and perform as intended. These regulations involve detailed documentation of product development and testing, with an emphasis on safety. To achieve CE (Conformité Européenne) or UKCA (UK Conformity Assessed) marking, technical documentation on safety and performance must be submitted; however, these data do not necessarily prove clinical efficacy. Robust validation of clinical performance may still be required.

The AoA Safety Guideline 2009, NAP 4, and DAS^9^^,^^30^^,^^31^ have made recommendations about the introduction and management of medical devices. These include equipment risk assessment, evaluation and procurement, user training, maintenance, disposal/replacement, and reporting of any adverse incidents. These processes are generally managed locally and can vary between organisations.

Procedures regarding research are more standardised and a detailed outline of these processes is beyond the scope of this document. In the UK, this requires application through the Integrated Research Application System (IRAS), approval by a Research Ethics Committee (REC), Health Research Authority (HRA), and the relevant research and development (R&D) department. In 2011, DAS launched the Airway Device Evaluation Project Team (ADEPT) with the aims to standardise and monitor new device evaluation and deliver evidence on safety and efficacy before widespread use.^31^ Sadly, ADEPT has struggled to engage industry, who continue, perhaps not unreasonably, to explore simpler, quicker, and cheaper ways to get their products evaluated and into clinical practice.

### Recommended practice

The ethical introduction of a new or existing CE/UKCA marked airway device into practice should start with evaluation of available performance and safety data, and then follow a locally agreed process that includes training, consideration of implications on consent, and the potential for collecting and sharing local performance data as part of post implementation surveillance. This is essential where performance data do not already exist.

The nature and extent of the evaluation will be influenced by the degree to which the airway device is considered novel and the level of complexity associated with its use. For a structurally familiar, simple, or ‘low tech’ device, an informal, local evaluation may suffice to ensure implementation into wider departmental practice is both safe and successful. Opportunities to collaborate with procurement teams and clinicians in other centres should be explored. Procurement will need to know the rationale for the proposal and may push for any change to be ‘cost neutral’. They may also insist on traceability of the new device as it progresses through the evaluation process.

For introduction of more complex, ‘high tech’ airway devices or devices that require a distinct change in practice, it is likely that the governance framework will stipulate the need for evidence of efficacy and safety from an approved research project. If a research study is not deemed necessary—and therefore not reviewed as part of the research ethics process—then local governance committee opinion should be sought to ensure adequate oversight of the project.

The training needs of the department must be evaluated when proposing a new method of airway management. Where a department, local experts, or governance teams agree that no additional skills are required, then the airway device can safely be introduced with the provision of product information to users. A simple example is a supraglottic airway device, similar to existing devices except in a minor way, perhaps colour or texture, that requires no change in practice. Allowing the opinion of a single individual to make this judgement is best avoided, as this is open to significant bias.

More complex or less familiar methods require training to ensure safe practice. This may, for example, be the case for a supraglottic airway device that requires a different insertion technique or has different weight bands for size selection or one that has unique performance characteristics. Decision making should be guided by departmental discussion, with input from the RCoA-DAS Airway Lead, the Training Programme Director, and those in the department with responsibility for equipment and governance.

The specific training requirements will vary considerably between devices, techniques, and individuals. It is likely that multimodal training will prove most effective. This might include departmental communication and manikin-based training. It may be followed by opportunity for expert-supervised clinical use or for further manikin practice as required. Manufacturers may provide training materials, either online or in person. More complex or completely novel devices may require a coordinated educational process across the whole airway team, with a record of competency assessment which can be included in clinicians' appraisal records. Effective implementation is key to safety. The AoA stipulates that ‘anaesthetists must be trained in the use of all the equipment that they may use’.^31^ It is the ethical responsibility of the individual clinician to ensure that they have received the appropriate training for any equipment they use and refuse devices where inadequate training has been delivered. Departments must provide training, time, and resources to allow this.^32^ After initial training, users must maintain new skills, so departments must consider how this will be achieved. Individual clinicians might decide on an appropriate usage frequency to maintain competency. They can assess the degree to which the new airway device has become routine in their practice and hence decide the need for patient discussion or the use of risk mitigation strategies.

When considering a new airway *technique* involving current devices, existing recommendations for ethical practice are less clear. This appears also to be true for NHS policy governing surgical technique innovation in the UK.^33^ Formal New Procedure Committees exist in some trusts, and these may allow for more objective decisions on adoption. Where these are not available, local departments must take responsibility for risk assessment and deployment planning. Novel airway techniques are largely proposed by clinicians rather than industry. However, the ethical principles underlying the introduction of a novel technique will be the same as for a device and so a consistent approach is required.

A technique that is largely familiar to an anaesthetist would generally not raise major ethical issues. Examples of such might include using a modified technique for insertion of a familiar supraglottic airway device or an intubation aid (bougie or stylet). More complex or novel techniques require demonstrable expertise to protect patients from potential harm. This will be true even of expert airway practitioners where a new technique can involve modification of a familiar skill to manage a specific or uncommon clinical challenge. Any perceived additional risks need careful consideration and may merit discussion with other local or distant expert colleagues to ensure that the proposed technique's benefits outweigh potential risks. Identified risks should be mitigated as far as possible. This risk–benefit assessment should be discussed with the patient during the consent process.

Where recognised advanced airway techniques are being introduced by individuals or teams less familiar with the approach—for example, use of specialist ‘tubeless field’ techniques, such as high-pressure source ventilation or high-flow nasal oxygenation—clinicians and departments should reflect on their ability to provide these techniques safely and seek assistance as appropriate.

As with the need for training, the level of patient information required for the proposed use of a new airway method depends on several factors. Device complexity, its perceived invasiveness, operator familiarity/competence, and the level of detail the patient wishes to receive are all relevant. Use of novel methods requires clear documentation on the content of consent discussion.

Finally, clinicians should be open and transparent about the sources of funding for the introduction or evaluation of any new technique or equipment. There is a potential risk of a conflict of interests that should be declared at the outset of any new project.

**Fig 2:**
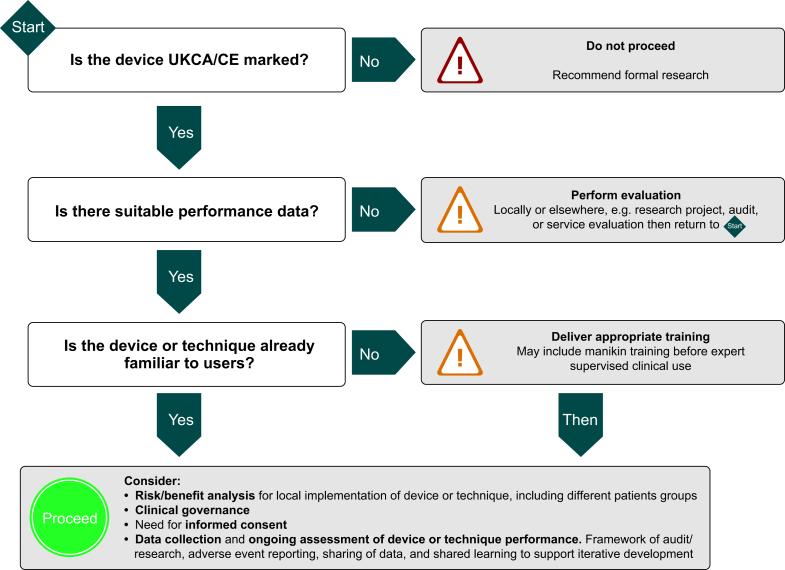
A flowchart on the steps recommended when introducing new airway devices or techniques. CE, Conformité Européenne; UKCA, UK Conformity Assessed.

**Fig 3:**
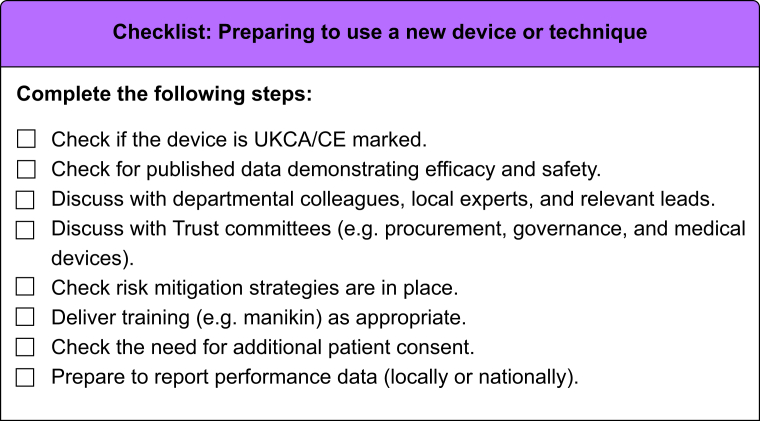
A checklist for preparing to introduce a new airway device or technique. CE, Conformité Européenne; UKCA, UK Conformity Assessed.

In summary, the regulations on airway device certification are well defined, but do not guarantee clinical utility. Assessment of utility requires high-quality data collection and analysis. Regulations for airway technique innovation are lacking, but best ethical practice ensures that introduction of new methods is as safe as possible. We strongly recommend that clinicians openly discuss proposals with local colleagues and consult Airway, Equipment, and Governance Leads. A plan to train all users is required, along with a clear risk assessment. Introduction should often occur within a framework of audit, service evaluation, or research, with a plan for collection of performance data and sharing of findings. A flowchart and a checklist on the steps recommended when introducing new airway devices or techniques are shown in Fig 2, Fig 3.

## Choosing the airway technique as part of training

**Fig 4:**
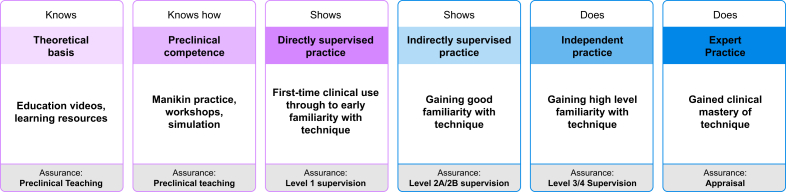
Stepwise progression of the steps associated with learning a new skill and how they relate also to the Royal College of Anaesthetists' designated levels of supervision.

Clinicians acquiring new skills do so incrementally. This stepwise progression (Fig. 4) can be seen in ‘Miller's Pyramid’, the base of which is formed by the ‘knows’ phase, progressing through ‘knows how’, ‘shows’, and finally ‘does’.^34^ In essence, this describes gaining a theoretical appreciation of the procedure sufficient to allow application of knowledge to a clinical problem. Learners then demonstrate to supervisors that they can perform in practice, first with close supervision and later with more independence as skills grow. Expertise is gained, and maintained, by repeated and deliberate practice.

Choosing an airway technique based on educational benefits requires careful consideration, particularly if there are no discernible advantages to the patient. Consideration of risks, relating either to the technique itself or the familiarity of the operator, is essential. Supervisors must minimise such risks by ensuring learners are ready to perform the method on patients. Evidence of progression through the stages highlighted in Fig. 4, whereby the doctor in training (hence referred to as a ‘trainee’) develops through non-clinical and clinical teaching stages, will satisfy this need. Figure 4 also highlights how these stages relate to the RCoA designated levels of supervision.^35^

The ethics of airway training could be considered a continuum, ranging from ethically straightforward to complex. This largely relates to how ‘routine’ the device or technique can be considered and on the risks and benefits involved. To provide some examples of this continuum, we offer categorisation of airway teaching situations that may be encountered:

(i) Minimal ethical issues: the airway technique has been specifically chosen for this patient regardless of educational benefit.
Even if the learner were not present, this technique would have been chosen—for example, the trainee using a hyperangulated videolaryngoscope (HAVL) to intubate a patient in whom difficulty is anticipated if a more conventional Macintosh-shaped device had been selected. Airway management will still provide significant educational benefit.
(ii) *Intermediate ethical issues: The airway technique is one of several (routine) options available to this operator, but the method selected is for educational benefits.*
The supervisor could choose one of many methods to manage the airway but has selected the one of maximal training benefit. An example of this could be the use of a HAVL purely for training benefit in a patient in whom difficulty with conventional Macintosh VL or DL is *not* anticipated; hence, there is no overall benefit to the patient from this choice. To ensure best ethical practice, the trainer should be able to justify the choice of technique as equivalent to alternatives in terms of risk and that it is ‘routine’ for them (i.e. they use it regularly). In addition, the trainee should be sufficiently familiar with the device in terms of the key principles in its use and should have practised using the device on manikins. Similarly, a less experienced trainer, choosing a device to acquire greater *personal* skill, should also have had training with the device and should improve this experience in low airway risk cases, with risk mitigation in place, until such skill with the device has been acquired that it can be considered routine practice.
(iii) Complex ethical issues: The choice of airway method has been made for educational benefit and would be considered as non-routine by most airway providers.
The modified technique chosen, still with the same endpoint (e.g. oral intubation), should be equivalent to alternatives in terms of potential risk to the patient, but this may be more difficult to quantify than in category 2. For example, use of supervised ‘asleep’ flexible bronchoscopic intubation, either directly or via a supraglottic device as a conduit, in a patient in whom the trainer anticipates no difficulty, will be considered acceptable by many clinicians. Consideration should be given to predictable risks, such as hypoxaemia from prolonged apnoea, and how these balance against potential educational benefits. Mitigation strategies listed in Table 2 should be used. *Awake* tracheal intubation purely for educational benefits will be considered an unreasonable option by some, but others will consider it reasonable subject to documented informed consent.^36^
(iv) *Ethically controversial: Choosing a different airway endpoint, purely for educational benefit or a technique where the risks outweigh the benefits.*
Choosing an airway approach with perceived greater risks, such as DL in a known difficult DL scenario, primarily to benefit training, would be controversial and, to most, unethical. A situation where a different, non-routine, airway endpoint is chosen purely for educational value is also ethically more challenging. An example would be use of a ‘tubeless field’ utilising trans-tracheal (cricothyroid cannula) high-pressure source (jet) ventilation in a laryngological procedure where the surgeon has stated that the surgery can be performed with a tracheal tube *in situ.* In all such ‘non-routine’ airway approaches, but particularly when a different endpoint has been chosen for training, there is a need for robust procedural governance, which includes detailed patient information, and a consent process that should occur before the immediate preoperative visit, providing time for consideration. The absence of informed consent when providing such training fails to respect patient autonomy and may constitute a form of harm.^37^

### Videolaryngoscopy

Tracheal intubation is the most discussed and debated airway intervention in anaesthesia. Historically, this procedure has been achieved using DL. Over recent years, however, VL has become increasingly used as a superior approach.^26^^,^^27^ Furthermore, a strong case can be made for using Macintosh VL to teach Macintosh DL as it facilitates better supervisor visibility of the learner's laryngoscopic view and better appraisal and optimisation of the learner's DL technique (operator is only using DL; teacher is using VL).^38^ Arguably, having the trainer use an indirect view, provided by a Macintosh VL, to enhance their supervision of a DL attempt by a learner, is the most *ethical* approach for novice intubation in clinical practice.

### A proposed ideal, ethically informed, airway training framework

Educational objectives should be clearly defined and agreed before the learning episode(s). Non-clinical learning, such as reading material, videos, or simulation (manikin, cadaveric, or virtual reality), should be completed beforehand whenever possible. A formalised training programme, such as the Mastery Learning approach, may facilitate this.^39^ The teaching should be appropriate to the learner's stage of training, with each exposure being tailored to the learner as they progress from baseline knowledge and skills to independent practice. More advanced techniques should be reserved for later stages of training when learners would be reasonably likely to require such a technique. Patients must be informed that both supervised and clinically appropriate training, as mentioned earlier, may be part of their care and they must freely consent to this.

As stated, the learner should also have a foreseeable use for the skill, and opportunities to maintain the skill after training. For example, medical students are required to learn basic airway management, but will not achieve or be able to maintain intubation skill after an anaesthesia attachment. It could be strongly argued that the risk–benefit balance would dissuade against teaching medical students intubation beyond their observing the procedure in much the same way as allowing an anaesthetic practitioner to intubate a patient, purely for their education, would be inappropriate.

When delivering training, trainers should seek feedback and reflect on their approaches as part of the *Recognition of Trainer* process at appraisal. This affords quality assurance of teaching methods.

**Fig 5:**
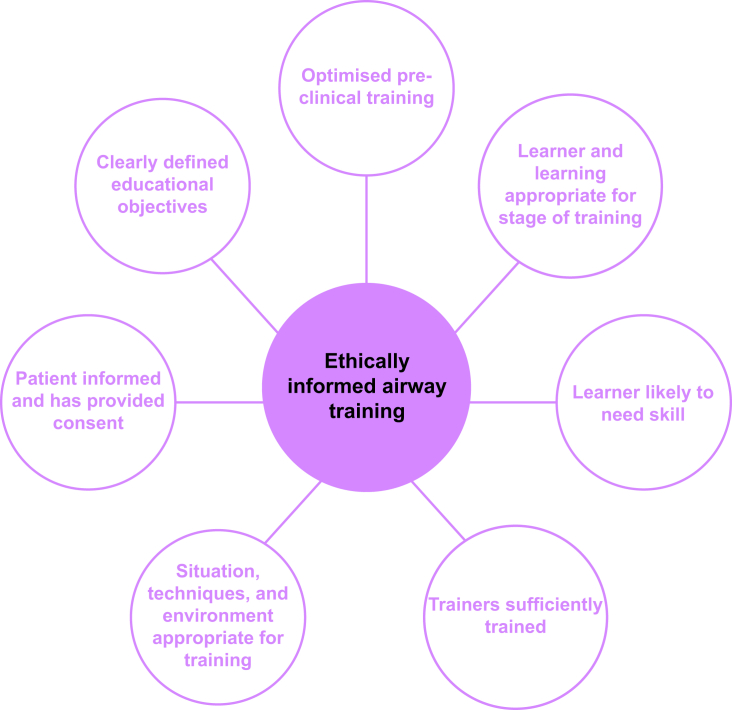
The key points of an ethically informed airway training framework.

In summary, the clinical situation, technique(s), and environment should all be appropriate for teaching. The patient should be fully informed and have given their consent to the procedure and the educational component. The key points are listed in Fig. 5.

## Patient consultation—information provision and communicating risk

The main aims in communicating on airway management are providing sufficient, meaningful information in a format that the patient can use. Anaesthetists must be aware of their own biases, thus avoiding coercion or undue patient stress while empowering patient choice. Establishing what level of information a particular patient needs and wants in the context of a first preoperative meeting within a limited timeframe means this can be challenging.

### Considering the views of patients

During interactions with the PatientsVoices@RCoA, guided discussions were focused on clinician engagement with patients with a distinction being made between pre-assessment clinic (PAC) and day-of-surgery consultations. The patient group reflected that PAC attendances were more suitable to detailed consideration of consent. These conversations could more easily be supported by printed or online patient information from reputable sources, such as the RCoA-DAS document *Your Airway & Breathing*.^40^ On the day of surgery, the group considered that patients are more anxious about the upcoming surgery and look for clinician positivity regarding anaesthesia and airway management. A detailed discussion over the risks of airway management is more difficult at this stage.

Three main themes emerged from the PatientsVoices@RCoA group relating to consent for airway management: trust, responsibility, and choice:

(i) Trust: The group consistently referred to the importance of trusting their clinician to make decisions in their best interests. Patient safety was stated as the priority; however, it was recognised that the educational needs of anaesthetists and the safety of future patients were also important and legitimate considerations.
(ii) Responsibility: The group thought that patients expect the clinician to be responsible for their airway management, probably without detailed discussion of the process at least for routine anaesthesia. They thought that patients would trust their anaesthetist to behave responsibly over airway management decisions, including training and the use of new methods. They accepted this may or may not involve patient discussion.
(iii) Choice: The group felt that patients would expect the clinician to assess the level of information and shared decision making appropriate for them individually. The group acknowledged that the extent of such conversations will vary between patients.

It should be noted that PatientsVoices@RCoA may represent patient views at one end of a spectrum in terms of levels of informed and interested opinion. It may not be representative of patients who are less familiar with anaesthesia, or patients who are less trusting of healthcare professionals.

### A suggested basic structure for a meaningful dialogue at the preoperative visit

The following is a potential framework for discussing airway management with patients, formulated by the authors and informed by input from PatientsVoices@RCoA. These conversations should complement those that should have occurred during pre-assessment.

(i) The first task is to ascertain that the patient has had access to approved patient information after operation that includes airway management. The RCoA-DAS document *Your Airway and Breathing During Anaesthesia*, updated in 2023, is an example of written information.^40^ Provision of this material well in advance of anaesthesia is best practice. It must be accepted that not all patients will read such material. However, failure to provide this at all will create a need to impart more information on the day. This is not ideal as it allows less time and provides a less suitable environment for the patient to process the information and allow personal consideration of material risk. In providing any information, clinicians must disclose important material risks but equally not bombard patients with excessive information or technical jargon. A careful balance must be struck such that the patient is informed but not confused nor overwhelmed.^14^
(ii) Share generic points about anaesthesia and airway management. This will often follow the sequence of events before, during, and after anaesthesia and airway management. Intubation and extubation are likely to merit specific mention. It might also provide an opportunity to outline aspects of airway teaching. Anaesthetists should recognise the importance of tailoring information to an individual.
(iii) *If the patient wishes*, progress to a more detailed discussion regarding specific details, options, and the consequences of each potential option. The information discussed should be proportionate to the risk of perceived difficulty or complexity of airway management.
(iv) Consider risk. Risk perception is highly subjective, with only the patient aware of their values, expectations, beliefs, and priorities.^41^ Use of the RCoA Common Events and Risks in Anaesthesia infographic (or equivalent) is recommended to aid risk discussion.^42^ Relative risk can be a complex concept to convey and use of a visual aid converting incidences into commonly understood frequencies people can relate to can help significantly. It is important to respect that patients may not wish to discuss risk in the immediate preoperative period.
(v) Documentation of what has been discussed is recommended and should include any specific aspects relating to that patient's airway management, such as particular risks relating to their situation or specific concerns that they may have.

When a patient lacks capacity, this structured approach can be used to support discussions with the patient's family or someone close to them.

### The right setting for patient conversations

It is important that the environment in which conversations are held is conducive to sharing information, shared decision making, and gaining informed consent. Ideally, the pre-assessment staff will have shared the relevant information, including the RCoA-DAS material, and discussed airway management, but not all elective patients will have an opportunity to attend pre-assessment, and many will not have had meaningful discussion regarding their airway management. Conditions in admission areas may cause difficulties; for example, they may be busy, noisy, or cramped. Clearly, in some emergency clinical situations, discussion with the patient may be brief or impossible. Even so, the clinician must share as much information as is necessary.

### Avoiding overburdening patients with information

It is possible to overburden the patient and create psychological distress, particularly when discussing complex concepts or serious complications. This was addressed in the Montgomery judgement.^14^ Excessive information can create a *nocebo effect*,^43^ where the patient suffers detriment from the interaction. GMC guidance states that one ‘must give patients any information they want or need about the potential risks, including the well-established complications of the procedure and any risk of serious harm, however unlikely it is to occur’,^2^ whereas the AoA cautions against overwhelming the patient with overly technical information they cannot comprehend.^26^ Ultimately, the extent of the discussion should be guided by the patient's communicated desires.

## Limitations

The subject of airway management ethics does not lend itself to using quantitative research evidence and meta-analysis. Therefore, we did not perform a formal systematic review, but drew on our initial scoping review of the relevant literature. The recommendations are based on expert opinion from a range of clinicians and academics, with input from patient representatives, but wider patient and public engagement was not included. Research exploring the attitudes and views of patients on the issues raised in this document would strengthen any future work.

This document, and the recommendations within it, is centred around UK anaesthetic practice but may still have relevance in other jurisdictions and in other specialties who provide airway management.

## Conclusions

**Fig 6:**
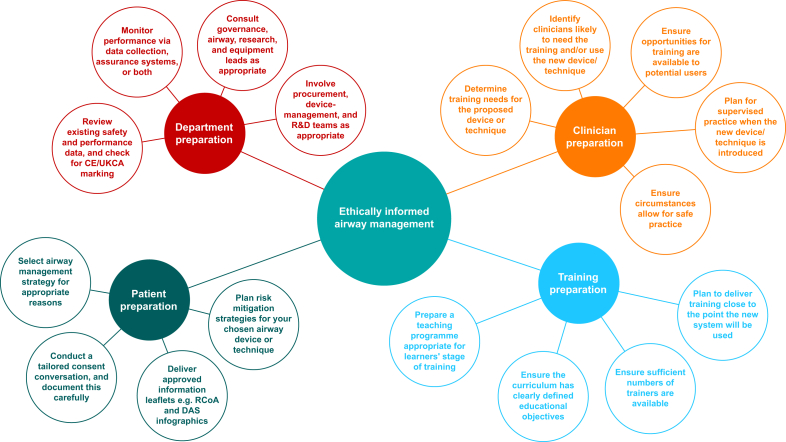
Multi-faceted considerations for ethically informed airway management. CE, Conformité Européenne; DAS, Difficult Airway Society; R&D, research and development; RCoA, Royal College of Anaesthetists; UKCA, UK Conformity Assessed.

This document is not prescriptive. The subject of ethics in airway management is broad and its delivery requires a multi-faceted approach (as summarised in Fig. 6). The recommendations are not based on empirical research evidence used in clinical airway management guidelines, rather on reasoning and expert opinion. Practitioners involved in airway management should reflect on and balance ethical issues in their practice, prioritising the quality of the current patient's airway management whilst considering benefits to team members and future patients. We hope this position statement and its recommendations may assist in this process in an ethical and social landscape that is continually changing.

## Declaration of interest

The authors declare that they have no conflicts of interest.
